# Draft Genome Sequence of the Yeast *Saccharomyces cerevisiae* GUJ105 From Gujarat, India

**DOI:** 10.1128/genomeA.01315-16

**Published:** 2016-12-01

**Authors:** Chandan Badapanda, Rajesh Detroja, Ankita Rathore

**Affiliations:** Xcelris Labs Limited, Ahmedabad, Gujarat, India

## Abstract

Here, we report the draft genome sequence of *Saccharomyces cerevisiae* strain GUJ105, isolated clinically. The size of the genome is approximately 11.5 Mb and contains 5,447 protein-coding genes.

## GENOME ANNOUNCEMENT

*Saccharomyces cerevisiae* is the first eukaryote for which a genome was sequenced (1). *S. cerevisiae* has been studied extensively for decades as a model organism for the following reasons: (i) it is used as a host for recombinant protein production (2), (ii) it is tolerant of a wide range of physiological stresses, such as low pH, high ethanol, and osmotic pressure (3, 4), (iii) it is used in the baking industry and in fermentations of food-grade alcohol production (5, 6), (iv) it is used as a model for biofuel production (7), (v) it is used as a model system for evolutionary biology (8), and (vi) it is used as a model for population and quantitative genetics. *S. cerevisiae* is found in multiple environments, one of which is the human body as an opportunistic pathogen (9, 10).

Here, we report the draft genome sequence of *S. cerevisiae*, derived from a clinical sample from Gujarat, India. The genomic DNA was sequenced using an Illumina HiSeq2500 platform. A total of 4.72 million paired-end reads having 2 × 150 bp chemistry with an estimated coverage of 62× were produced. The adapter sequences were trimmed from the reads before assembly using Trimmomatic (v0.30) (11). *De novo* assembly was carried out with the assembly tool Velvet (12) in conjunction with the VelvetOptimiser (k-mer 67) (http://bioinformatics.net.au/software.velvetoptimiser.shtml).

The resulting assembly has 201 scaffolds with a total length of 11,563,971 bases (11.5 MB) (the largest scaffold having a length of 552,249 bp), an *N*_50_ of 276,334, and a G+C content of 37.95% and 100% of completeness, as estimated by mapping the orthologous genes (KOG databases) using CEGMA (13). Using *S. cerevisae*, the gene finder Augustus (14) identified 5,447 protein-coding genes. An automatic annotation using the BLASTp algorithm (15) revealed 5,447 predicted proteins with significant similarity (*E* value cutoff = 10^−5^) to sequences deposited in the nonredundant (nr) protein database from NCBI. Using tRNAscan-SE version 1.3 software (16), we found 266 tRNA genes scattered across the scaffolds.

In our future study, we will carry out a comparative genomics study of strain GUJ105 with the existing clinical strains for better understanding of the basic mechanisms of adaptation to its natural habitat, as well as of its pathogenicity and virulence.

### Accession number(s).

Data related to the draft genome project of *Saccharomyces cerevisiae* GUJ105 have been deposited at GenBank under the accession number MCNH00000000.
